# Coral cover and rubble cryptofauna abundance and diversity at outplanted reefs in Okinawa, Japan

**DOI:** 10.7717/peerj.9185

**Published:** 2020-09-22

**Authors:** Piera Biondi, Giovanni Diego Masucci, James Davis Reimer

**Affiliations:** 1 Molecular Invertebrate Systematics and Ecology Laboratory, Graduate School of Engineering and Science, University of the Ryukyus, Nishihara, Okinawa, Japan; 2 University of the Ryukyus, Tropical Biosphere Research Center, Okinawa, Japan

**Keywords:** Coral reefs, Coral reef restoration, Coral rubble, Cryptofauna, Marine biodiversity, Okinawa Island, Japan

## Abstract

Global climate change is leading to damage and loss of coral reef ecosystems. On subtropical Okinawa Island in southwestern Japan, the prefectural government is working on coral reef restoration by outplanting coral colonies from family Acroporidae back to reefs after initially farming colonies inside protected nurseries. In order to establish a baseline for future comparisons, in this study we documented the current status of reefs undergoing outplanting at Okinawa Island, and nearby locations where no human manipulation has occurred. We examined three sites on the coast of Onna Village on the west coast of the island; each site included an outplanted and control location. We used (1) coral rubble sampling to measure and compare abundance and diversity of rubble cryptofauna; and (2) coral reef monitoring using Line Intercept Transects to track live coral coverage. Results showed that rubble shape had a positive correlation with the numbers of animals found within rubble themselves and may therefore constitute a reliable abundance predictor. Each outplanted location did not show differences with the corresponding control location in terms of rubble cryptofauna abundance, but outplanted locations had significantly lower coral coverage. Overall, differences between sites (Maeganeku1, Maeganeku2 and Manza, each including both outplanted and control locations) were significant, for both rubble cryptofauna and coral coverage. We recommend (1) to outplant colonies from more stress-resistant genera in place of *Acropora*, and (2) to conduct regular surveys to monitor the situation closely. With a lack of baseline data preceding impacts, rigorous monitoring over time can highlight trends towards increases or decreases in evaluated variables, allowing to obtain a clearer idea of the effects of transplants and on the trajectory of impacts due to climate change and local stressors . Finally, we also recommend (3) to establish conservation and sustainable practices that could aid the ongoing restoration efforts such as installing anchoring buoys to reduce impacts from anchoring, which could reduce coral mortality of both outplanted and native coral colonies.

## Introduction

Coral reefs in the Indo-Pacific are the most diverse marine ecosystems in the world (Hoeksema, 2007). In southwestern Japan, the Ryukyu Archipelago provides a favorable environment for more than 360 zooxanthellate scleractinian coral species (Nishihira, 2004). Such high levels of marine biodiversity are connected with the presence of the warm Kuroshio Current which flows northwards across the islands of the archipelago. Marine biodiversity in the Ryukyus, compared to other regions in the Indo-Pacific, such as the Red Sea or the Great Barrier Reef, is still relatively understudied (Fujii & Reimer, 2011; Reimer et al., 2019), and studies on ecological processes, conservation-related issues and human impacts are also comparatively few (Reimer et al., 2019).

Coral reefs in the Ryukyus are facing a decline due to a combination of temperature-induced bleaching caused by climate change (Nakano, 2004a) and local stressors, including overfishing, soil runoff, pollution, habitat loss and fragmentation (Nakano, 2004b; Hongo & Yamano, 2013; Reimer et al., 2015; Masucci & Reimer, 2019; Masucci et al., 2019; Reimer et al., 2019). The net result is a decline in overall ecosystem health; scleractinian corals have been facing reductions in both coverage and diversity, particularly branching coral genera such as *Acropora* and *Montipora*, once dominant in the Ryukyu Archipelago (Sakai & Nishihira, 1986; Mori, 1995; Loya et al., 2001; Omori, 2011; Van Woesik et al., 2011; Masucci et al., 2019).

### Coral outplanting and restoration efforts in Okinawa

**Figure 1:**
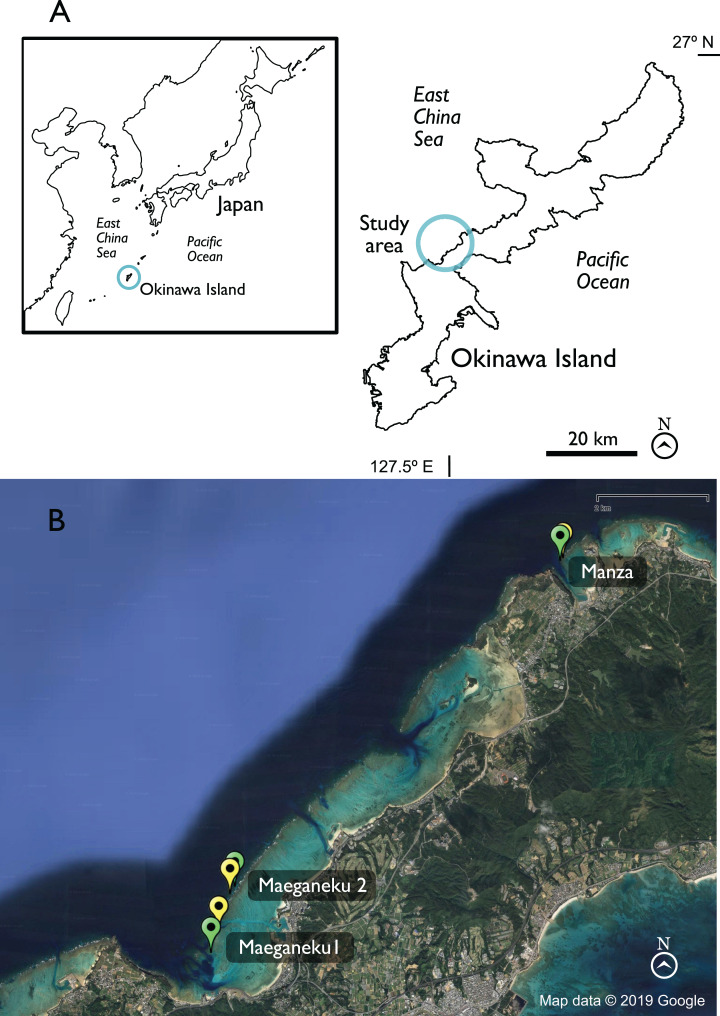
Map of Okinawa and study area. (A) General view of Okinawa. (B) Study sites in detail. Green = Control locations, yellow = Outplanted locations. Map data © 2019 Google.

The southern half of the Ryukyu Islands is administered by the Okinawa Prefectural Government (Okinawa Prefecture), and includes Okinawa Island, the largest and most populated island of the Ryukyus (area = 1,208 km^2^; Japan Statistics Bureau, 2014; population ∼1.25M people; Okinawa Prefectural Government, 2019). Ecological restoration is defined as the act of assisting a destroyed or degraded ecosystem in its healing process (SER, 2004). To mitigate the decline of local reefs, restoration attempts were initially carried out by local fishing unions and dive centers in the late 1990s (Okubo & Onuma, 2015). These first efforts were based on outplanting of colonies asexually generated by fragmenting donor colonies from nearby sites. Starting from 2011 restoration efforts have been coordinated and directed by the prefectural government (Omori et al., 2016). The actual outplanting of new coral colonies under prefectural supervision started in 2012 at Onna Village (Fig. 1A) on the west coast of Okinawa Main Island, where colonies are still being outplanted yearly, and is planned to be expanded in the future to the declining reefs of Kume Island (Masucci et al., 2019). In Onna, the project aims to produce approximately 20,000 colonies per year (Higa & Omori, 2014) with a target of over 40% survival rate after 3 years (Omori et al., 2016).

The restoration approach in Okinawa is based on the initial collection of fragments (~5 cm) from nearby reefs. Fragments are grown in protected lagoons on top of iron poles, about 50 cm above the seafloor, to be used as donor colonies. When fragments reach a size of about 30 cm, 60% of each donor colony is fragmented. These new fragments are fixed to hard substrates of various shapes (Omori et al., 2016). After growing in nurseries for a further 3 months, fragments that reach a size > 5 m cm are transferred to their final destination, the coral reef locations targeted for restoration (Higa & Omori, 2014; Omori et al., 2016). Using this method, about 20 species of Acroporidae, approximately 10,000 colonies per year, have been transplanted in the waters off Onna Village, at the cost of 2,000 Japanese yen (approx. 18 USD) per colony (Omori et al., 2016; Higa et al., 2018). Transplanting *Acropora* and branching corals is aimed at increasing spatial complexity, providing a higher number of available niches for coral reef organisms (Vytopil & Willis, 2001; Graham & Nash, 2013).

The purpose of this research was to assess the present status of the reefs at Onna Village, at coral outplanted and surrounding non-outplanted locations, in order to establish a baseline for future monitoring. Additionally we examined if the outplanting operations had short-term effects (e.g., within months to years since commencement of outplanting operations) on coral cover and cryptofauna abundance and diversity. For these reasons, in this study, in addition to traditionally used methods to assess the reef based on coral cover, we utilized an approach to quantify and compare benthic diversity using mobile cryptofauna communities.

Coral cryptofauna are composed of metazoan organisms living in inter- and intra-skeletal structures of hard corals, including dead corals and coral rubble (Enochs, 2011), and play an important role in maintaining coral reef functionality by capturing and recycling nutrients and providing biomass to upper trophic levels (Richter et al., 2001; Enochs, 2011; Kramer, Bellwood & Bellwood, 2013). Coral cryptofauna inhabit a variety of marine environments and have been used as proxies for benthic diversity in past studies (Takada, Abe & Shibuno, 2007; Enochs, 2011; Takada et al., 2014; Wee et al., 2019). Rubble cryptofaunal surveys can allow comparisons of the animal community living at each location in order to evaluate differences in biodiversity.

It has been proven that more spatially complex and larger substrates can host a higher number of cryptic animals (Kostylev et al., 2005; Fraser & Sedberry, 2008). Thus, it is possible that the areas of coral transplantation harbor a higher number of cryptofaunal animals than surrounding unaltered areas. We additionally analysed the correlation between rubble shapes and the abundance of rubble cryptofauna in order to test the hypothesis that cryptofauna abundance would be positively related with higher degrees of substrate complexity.

## Materials and Methods

### Sampling area and experimental design

Sampling was performed at three sites in Onna Village, the southern side of Maeganeku (hereafter Maeganeku1), the northern side of Maeganeku (hereafter Maeganeku2), and Manza. Each site consisted of a coral outplanted location and a nearby control location, where no outplanting operations had been conducted (Table 1; Fig. 1). We selected control locations in the proximity (~100–400 m) of their respective outplanted location, in order to have, comparable conditions within each site. Maeganeku1 (26.44715° N, 127.79077° E) was located at the south side of a dredged canal that connects Maeganeku Port to the outer reef. It featured an extended shallow fringing reef area (length >1 km; depth 2–4 m) followed by a slope that reaches 15 m of depth along a channel with a sandy bottom. At the end of the reef slope, the seafloor was characterized by an accumulation of coral rubble forming mounds with the frequent presence of discarded fishing nets, suggesting some commercial fishing pressure in the area. Maeganeku2 (26.453902° N, 127.79313° E) was located on the north side of the same channel, where the inner reef was slightly shorter (~800 m, with similar depth as Maeganeku1). The depth at Maeganeku2 increased along a gentle slope to more than 40 m in depth. Manza (26.508497° N, 127.85287° E) was located at the north side of Cape Manzamo. The Manza site is a popular diving spot known for its high coral cover, and relatively healthy conditions (no industrial activities, dredging or land reclamations in its proximities), although the presence of discarded fishing line suggested some recreational fishing pressure. Manza was characterized by a short fringing reef (length ~100 m, depth 3–10 m) followed by a relatively steep slope that goes down to 20–50 m to a sandy seafloor (Kamezaki et al., 2013). Outplanting of new colonies under the supervision of the Okinawa Prefectural Government started from 2012 at Maeganeku1 (which is where the local community had been transplanting colonies since the end of the 1990s), and from 2017 at Maeganeku2 and Manza (Okinawa Prefectural Government, 2017; Onna Village Fisheries Cooperative, 2017, personal communication).

**Table 1:** Latitude and longitude of locations in this study.

Location	Latitude and Longitude
Maeganeku Outplanted 1	26.44715, 127.79077
Maeganeku Control 1	26.443167, 127.788947
Maeganeku Outplanted 2	26.453902, 127.79313
Maeganeku Control 2	26.454056, 127.793351
Manza Outplanted	26.508497, 127.85287
Manza Control	26.506983, 127.85207

Fieldwork was performed in winter 2017, summer 2018 and winter 2019. Each season, we investigated the same three sites (for each site, one outplanted and one control location). Three buckets (10 liters each) of coral rubble were collected (total *n* = 3 buckets × 6 locations, 18 buckets per season) from each location by SCUBA diving at the same depths as transplanted colonies (between 2 and 4 m). Within each location, rubble sampling was replicated at different rubble accumulation areas more than 10 m apart. Immediately after rubble collection buckets were sealed with lids to prevent mobile animals from escaping. Buckets were then transported back to Maeganeku Port, with the sorting process starting immediately after returning (within an hour after the end of each dive).

Images of collected coral rubble were taken immediately after sampling (Fig. S1). The shape of each coral rubble fragment was then classified as “massive–submassive” or “branching–tabular” following the coral classification protocol as described in English, Wilkinson & Baker (1997), using the images and descriptions of “lifeform categories” for benthic organisms. For each survey, the frequency of “branching–tabular” of the total rubble was calculated and correlated with collected rubble animal abundance and phyla diversity data.

Each cryptofaunal specimen of size ≥1 mm was collected with tweezers or syringes and photographed with macro lenses (Sony SEL30M35 f/3.5 mounted on Sony a6x00 camera) and scales (Fig. S2). Initially each animal was identified to the phylum level, given a specimen number, and preserved in 99% ethanol.

Additionally, collected rubble data were compared with more traditionally utilized approaches: during winter 2019 sampling, coral coverage data were collected from all six locations via LIT (Line Intercept Transects; Beenaerts & Berghe, 2005) (10 m each, for each location *n* = 6 transects) at the same depth as outplanted coral colonies (2–4 m). Photographs were taken and analyzed to calculate the percentage of coral and rubble coverage. Living corals were identified to genus level following Veron (2000) except for families Merulinidae and Montastraeidae, for which we followed Huang et al. (2014).

### Statistical analyses

#### Coral coverage

Statistical tests were performed using R software (version 3.6.0, R Development Core Team, 2019). To verify the presence of significant differences in coral coverage between sites and/or treatments (outplanted vs. controls), a 2-way ANOVA was performed. Normality was tested with the Shapiro–Wilk test (Royston, 1982), and homoscedasticity with Bartlett’s test (Bartlett, 1937). Data were not normally distributed. Therefore, before proceeding with ANOVA, data were normalized using a log+x transformation. After transformation, all data respected the aforementioned ANOVA assumptions. The Tukey post-test (Yandell, 1997) was performed on significant ANOVA results. The same was done for the cover of genus *Acropora*. Other coral genera were not normally distributed, even after attempting data transformation. Differences in their coverages between outplanted and controls were therefore compared using a non-parametric Kruskal–Wallis test (Hollander, Wolfe & Chicken, 2013).

To test differences in the coverage of the whole coral community between different sites and treatments, PERMANOVA was performed using the *adonis* function from the *Vegan* package for R (version 2.5-2, Anderson, 2001) with a Bray-Curtis distance and 9999 permutations. To highlight correlation patterns between the coverage of different coral genera with Site and Treatment, Principal Component Analyses (PCA) were performed, using the *rda* function from the *Vegan* package for R (version 2.5-2, Anderson, 2001). Results were displayed as a biplot (scaling 2, Gabriel, 1971) with coral genera expressed as arrows and locations as labels.

#### Coral rubble

The effects Treatment (outplanted vs. control) and Site factors on animal abundance were tested using a 2-way ANOVA. Additionally, a 1-way ANOVA was conducted for data from winter 2019, comparing outplanted and control locations. Normality was tested with the Shapiro–Wilk test (Royston, 1982), and homoscedasticity with Bartlett’s test (Bartlett, 1937). Since both assumptions were respected, no transformation of data was performed. The Tukey post-test (Yandell, 1997) was performed on significant ANOVA results.

The effects of the Treatment and Site factors on phyla abundance were tested with PERMANOVA (*adonis* function, *Vegan* package for R) using Bray–Curtis distance and 9,999 permutations. Correlation between rubble shape and animal abundance was tested using the Kendall’s tau correlation coefficient (Kendall, 1938). To highlight correlation patterns between locations, phyla distribution and rubble shape Principal Component Analyses (PCA) were performed. Results were displayed as a biplot (scaling 2, Gabriel, 1971) with phyla and “branching–tabular” frequency expressed as arrows and locations as labels.

## Results

### Living coral community

**Figure 2:**
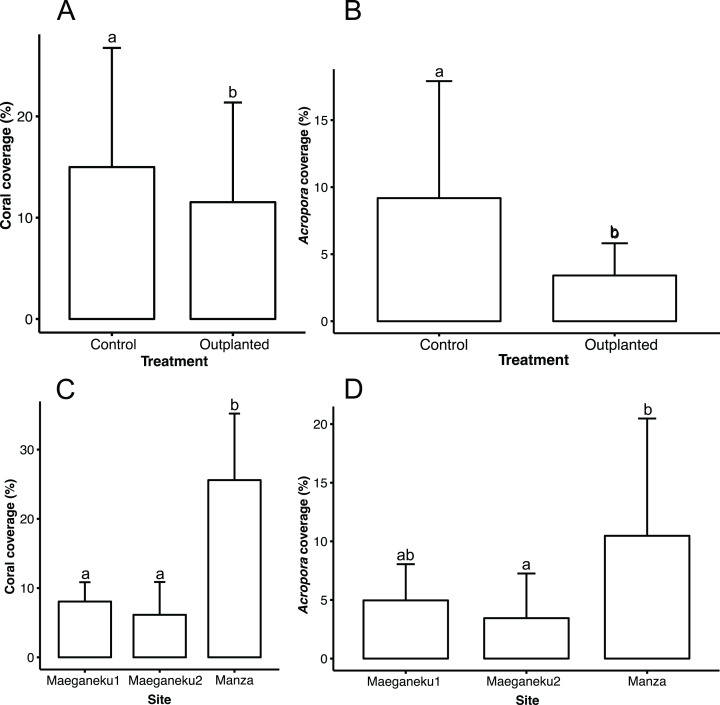
Mean coral cover (%) barplots. (A) Total coral cover at control and outplanted sites (2-way ANOVA; *F* = 4.487, *P* = 0.043). (B) *Acropora* cover at control and outplanted locations (2-way ANOVA; *F* = 12.813, *P* = 0.001). (C) Total coral cover at the three sites surveyed in this study (2-way ANOVA; *F* = 35.062, *P* < 0.001). (D) *Acropora* cover at the three sites surveyed in this study (2-way ANOVA; *F* = 6.758, *P* = 0.004). Error bars represent standard deviation, letters indicate statistical significances.

Manza was the site with highest living coral coverage (25.6% ± 9.6%), followed by Maeganeku1 (8.0% ± 2.8%) and Maeganeku2 (6.1% ± 4.7%). Differences in coverage between sites were statistically significant (2-way ANOVA; *F* = 35.062, *P* < 0.001). Manza differed significantly from Maeganeku1 (Tukey post-test; *P* < 0.001) and Maeganeku2 (Tukey post-test; *P* < 0.001) (Fig. 2), while differences between Maeganeku1 and Maeganeku2 were not significant (Tukey post-test; *P* = 0.153). Manza also had the highest coverage (10.5% ± 10%) of *Acropora*, the target genus being actively transplanted, followed by Maeganeku1 (5.0% ± 3.0%) and Maeganeku2 (3.5% ± 3.8%), and these differences were statistically significant (2-way ANOVA; *F* = 6.758, *P* = 0.004) (Fig. 2).

Control locations showed a higher living coral coverage (15.0% ± 11.8%) than outplanted locations (11.5% ± 9.8%), overall (Fig. 2), and for each single site (Table 2). The difference in coral cover between outplanted and control locations was statistically significant (2-way ANOVA; *F* = 4.487, *P* = 0.043). In a similar way, *Acropora* had significantly higher coverage at control locations (9.2% ± 8.7%) than at outplanted locations (3.4% ± 2.4%), overall (Fig. 2), and for each single site (Table 2). The observed difference in the cover of *Acropora* was statistically significant (2-way ANOVA; *F* = 12.813, *P* = 0.001).

**Table 2:** Coral cover and *Acropora* cover for each locations.

Site	Location	Coral cover (mean) (%)	Coral cover (SD) (%)	*Acropora* (mean) (%)	*Acropora* (SD) (%)
Maeganeku1	Control	9.2	2.1	6.4	3.1
Maeganeku1	Outplanted	7.0	3.1	3.5	2.4
Maeganeku2	Control	8.0	5.8	5.1	5.0
Maeganeku2	Outplanted	4.3	2.7	1.8	0.6
Manza	Control	27.9	11.5	16.0	11.8
Manza	Outplanted	23.3	7.6	5.0	2.7

**Figure 3:**
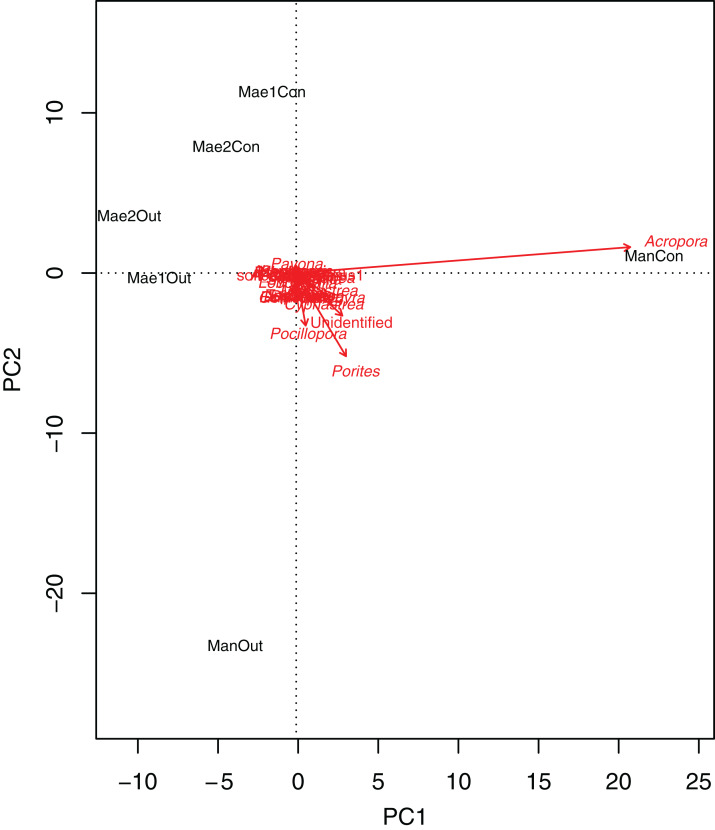
Principal component analysis biplot showing correlation patterns between the coverage of different coral genera and Site and Treatment. Corals genera expressed as arrows and locations as labels. Mae1Con = Maeganeku1 Control; Mae1Out = Maeganeku1 Outplanted; Mae2Con = Maeganeku2 Control; Mae2Out = Maeganeku2 Outplanted; ManCon = Manza Control; ManOut = ManzaOutplanted.

Generally, the coral community differed significantly between treatments (2-way PERMANOVA; R^2^ = 0.06377, *P* = 0.011) and sites (2-way PERMANOVA; R^2^ = 0.15775, *P* < 0.001). The PCA biplot (Fig. 3) highlighted that *Acropora* and the control location at Manza were strongly associated, with other associations between locations and coral genera being weaker. Although several scleractinian coral genera showed higher mean coral coverages at outplanted locations (Table 3), differences were not statistically significant (Kruskal–Wallis test; *P* > 0.05).

**Table 3:** Coral coverage of different coral genera in control versus outplanted locations. Asterisks indicate significant differences.

Genus	Control (%)	Outplanted (%)
*Achantastrea*	0.00	0.02
Acropora*	9.18	3.41
*Astreopora*	0.38	0.27
*Coeloseris*	0.06	0.00
*Cyphastrea*	0.52	0.44
*Echinopora*	0.04	0.28
*Dipsastrea*	0.42	0.59
*Favites*	0.38	0.48
*Galaxea*	0.00	0.03
*Goniastrea*	0.33	0.18
*Goniopora*	0.00	0.28
*Leptoria*	0.04	0.34
*Leptoseris*	0.02	0.00
*Lobophyllia*	0.02	0.12
*Montastrea*	0.32	0.29
*Montipora*	0.01	0.19
*Pachyseris*	0.00	0.04
*Pavona*	0.22	0.00
*Platygyra*	0.47	0.34
*Pocillopora*	0.31	0.80
*Porites*	1.41	2.34
*Psammocora*	0.03	0.00
*Symphyllia*	0.09	0.09
*Turbinaria*	0.01	0.24
Unidentified	0.74	0.66

### Rubble cover and shapes

Mean rubble cover was 2.3% ± 6.3%, with no differences between different sites (2-way ANOVA; *F* = 0.004; *P* = 0.949) or between outplanted and restored locations (2-way ANOVA; *F* = 1.880; *P* = 0.170).

Maeganeku1 was rich in “branching–tabular” rubble (84.3% of all rubble sampled at this site), with “massive–submassive” shapes being a minority of the total (15.7%). Conversely, at Maeganeku2, the frequency of “branching–tabular” rubble was only 7.7%, with most rubble having a “massive–submassive” shape (92.3%). Manza displayed an intermediate situation, with 35.5% “branching–tabular” rubble and 64.5% “massive–submassive”.

The aforementioned differences among sites were significant (2-way ANOVA; *F* = 11.490; *P* = 0.002), but there were no significant differences between outplanted locations and controls (2-way ANOVA; *F* = 0.380, *P* = 0.549).

**Figure 4:**
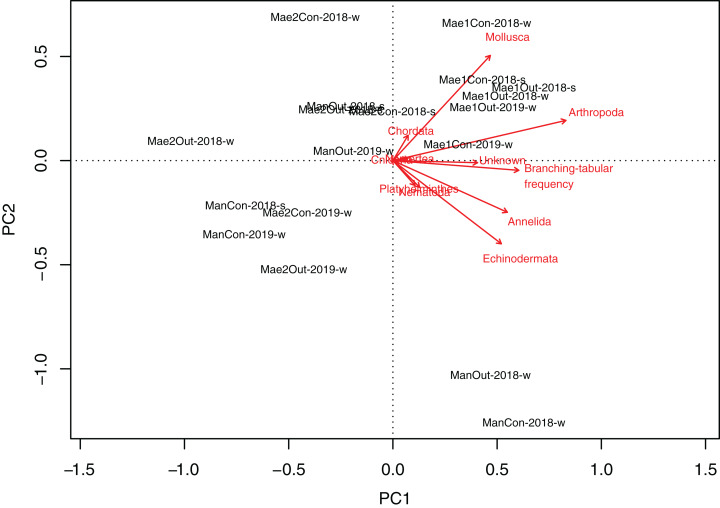
Principal Component Analyses biplot showing correlation between rubble shape and animal abundance at different locations. Phyla and “branching–tabular” frequency expressed as arrows and locations as labels.

Maeganeku1 was significantly different from Maeganeku2 (Tukey post-test; *P* = 0.001) and Manza (Tukey post-test; *P* = 0.027), but differences were not significant between Maeganeku2 and Manza (Tukey post-test; *P* = 0.237). Indeed, there was a strong positive correlation between the frequency of “branching–tabular” rubble and total abundance of rubble mobile cryptofauna (Kendall’s tau correlation coefficient; tau = 0.64; *P* < 0.001). The PCA biplot showed how rubble shape affected abundances within phyla (Fig. 4): Maeganeku1, richer in “branching–tabular” rubble, was associated with higher levels of abundances for all phyla. Conversely, Maeganeku2, characterized by “massive–submassive” rubble shapes, showed less total abundance and lower abundances within individual phyla.

### Rubble cryptofauna

#### Total abundance

**Figure 5:**
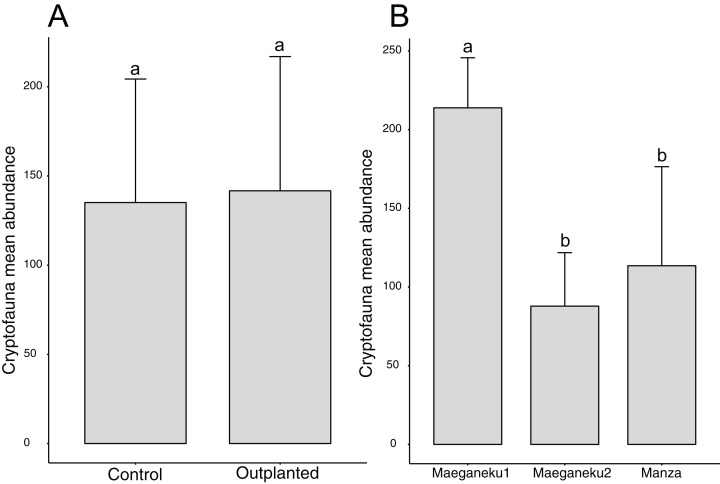
Mean animal abundances barplots. Number of animals per (A) treatment and (B) sites (2-way ANOVA; *F* = 12.3, *P* < 0.001). Error bars represent standard deviation, letters indicate statistical significances.

A total of 2,491 specimens were collected from coral rubble. Arthropoda was the most abundant phylum with 1,272 specimens, followed by Mollusca (591) and Annelida (235). Maeganeku1 showed the highest abundance of animals with 1,283 specimens, 685 in the outplanted location and 598 in the control location. Manza followed with a total of 681 specimens, 384 in the outplanted location and 297 in the control location. Maeganeku2 was the least numerous in terms of animal abundance (*n* = 527) with 206 in the outplanted location and 321 in the control location. Sites had a significant effect on cryptofauna abundance (2-way ANOVA; *F* = 12.3, *P* < 0.001) (Fig. 5). Following the same pattern observed for differences in rubble shape frequencies, differences were driven by Maeganeku1, which was significantly different from Maeganeku2 (Tukey post-test; *P* = 0.001) and Manza (Tukey post-test; *P* = 0.007), while Manza and Maeganeku2 did not show any significant differences (Tukey post-test; *P* = 0.617). Conversely, Treatment (outplanted vs. controls) did not significantly affect total cryptofauna abundance (2-way ANOVA; *F* = 0.090, *P* = 0.770) (Fig. 5). The effect of treatment was also not significant when analyzing data within the same season for both winter (1-way ANOVA; *F* = 0.002; *P* = 0.960) and summer (1-way ANOVA; *F* = 0.105; *P* = 0.760), or by analyzing the single winter 2019 dataset (most recent data points. 1-way ANOVA; *F* = 0.428; *P* = 0.549).

#### Abundances within phyla

**Figure 6:**
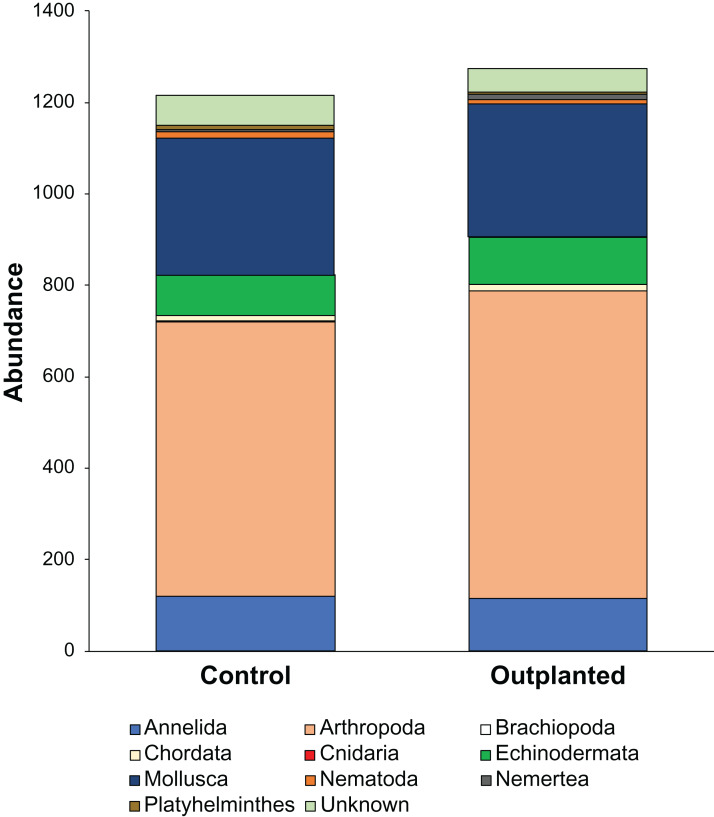
Abundances of different phyla within treatments.

Maeganeku1 was the most abundant site for Annelida, Arthropoda, Chordata and Nemertea. This location also had the highest number of unidentified specimens. Maeganeku2 had the lowest number of phyla (6 out of 9) and also had the lowest number of unidentified specimens. However, it was the only site where Brachiopoda were found. Manza was the most numerous for Echinodermata, Nematoda and Platyhelminthes. Differences in communities between sites were statistically significant (2-way PERMANOVA; total df = 17, *R*^2^ = 0.37, *P* = 0.003) (Table 4), while no differences emerge between outplanted locations and controls (2-way PERMANOVA; total df = 17, *R*^2^ = 0.019, *P* = 0.783) (Fig. 6). Even within the same season, there were no significant differences between outplanted and control locations, for both winter (1-way PERMANOVA; df = 11; *R*^2^ = 0.022; *P* = 0.921) and summer (1-way PERMANOVA; df = 5; *R*^2^ = 0.107; *P* = 0.894).

**Table 4:** Mean phyla abundances per site (combined treatment and control) in this study.

	Maeganeku1		Maeganeku2		Manza	
	Mean	SD	Mean	SD	Mean	SD
Annelida	16.2	7.8	8.2	5.5	14.8	17.0
Arthropoda	121.0	26.5	39.5	20.2	51.5	20.7
Brachiopoda	0.0	0.0	0.7	1.0	0.0	0.0
Chordata	2.3	2.2	1.2	1.2	0.5	1.2
Echinodermata	11.2	4.4	4.3	3.0	16.7	19.0
Mollusca	49.3	10.8	29.5	20.7	19.7	9.8
Nematoda	1.2	1.2	0.2	0.4	2.3	3.4
Nemertea	1.3	1.9	0.0	0.0	1.2	0.8
Platyhelminthes	0.8	0.4	0.2	0.4	1.7	3.1
Unidentified	10.5	6.7	4.0	2.8	5.2	5.4

## Discussion

### Living coral community

Control locations had a significantly higher living coral coverage and *Acropora* coverage, meaning that the outplanting operations did not lead to a direct increase in the target genus *Acropora*. However, outplanting is the initial phase of a longer process, whose benefits in terms of recovery may take multiple years and even decades (Pearson, 1981; Connell, Hughes & Wallace, 1997; Graham et al., 2015), and thus could not be assessed at the present time except for Maeganeku1, where coral transplantation has been conducted across two decades. For hard corals, the juvenile growth phase has been shown to be slow (Wallace, 1983; Harriott, 1985; Babcock, 1985) and connectivity to be a key issue, especially for broadcast spawners (Gilmour et al., 2013). *Acropora*, considered a fast-growing genus, has shown full recovery in natural conditions in about 7 years across several locations (Van Woesik et al., 2011). Actively outplanting colonies might lead to faster recovery times than just waiting for natural recovery to occur (Soong & Chen, 2003), but the timing of recovery is highly variable. Overall, with low coral coverages of ~8% (Maeganeku1) and ~6% (Maeganeku2), the coral reefs at Maeganeku are not in a healthy state.

Quantitative baseline data of the situation back to when the outplant efforts where started would certainly help in quantifying changes over time as well as to track more precisely the outcome of outplanted locations. Unfortunately, these data are not available, and were apparently never taken. However, there is qualitative information stating that the whole reef in front of Maeganeku (which includes both outplanted and control locations) at the start of the project was in a similar state of degradation (Okinawa Prefectural Government, 2017). The data provided by our work can be used as a baseline for future comparisons on the trajectory of these reefs, including potential eventual recovery.

Outplanting operations can sometimes harm the pre-existing community (Edwards & Clark, 1999; Casey, Connolly & Ainsworth, 2015). Branching corals are comparatively fragile and can be easily broken during transplantation activities. In the sites of this study, boats do not employ buoys and are secured by anchoring on the seafloor, including during restoration and monitoring activities. Fins, boots, and drilling holes in the reef during outplanting (see images 2.2. 1–10 and 2.2. 1–12 in Okinawa Prefectural Government, 2017) may also damage pre-existing corals, with control locations spared from these effects. An excessive accumulation of coral rubble can also impact living coral colonies (Rasser & Riegl, 2002; Masucci et al., 2019). This does not seem to be the case of this study, as there were no significant differences in rubble coverage (<5% for all locations) between treated and control locations. However, as rubble are not fixed to the substrate, and storms can potentially relocate them down the reef slope, it is possible that the present situation is not reflective of damage done by rubble in past years. The relevance of these issues along the west coast of Okinawa Island should be assessed in the future via rigorous pre-outplanting assessment combined with detailed monitoring of sites.

Compared to our results, the situation may have been different before 2016, as survival rates for colonies transplanted at Maeganeku1 after 2013 was reported to be exceeding the expected performance targets (>40% at 3 years after transplantation; Omori et al., 2016). Subsequently, and before the current study, in the summers of 2016 and 2017, the Ryukyu Islands experienced two consecutive bleaching events, which had serious negative consequences on shallow water hard corals across the archipelago, particularly for branching/tabular species (Kayanne, Suzuki & Liu, 2017; Masucci et al., 2019).

### Rubble cryptofauna

The coral transplantations did not have any observable significant effect on rubble cryptofauna, both in terms of numbers and phyla diversity. Conversely, sites significantly affected both cryptofauna abundance and its diversity at the phylum level. This could be explained by differences in physical parameters (such as rubble size and shape) (Troyer, Coker & Berumen, 2018, but see Takada et al., 2014), as well as succession (Choi, 1984), wave action (Kobluk & Lysenko, 1987), fish abundance (Troyer, Coker & Berumen, 2018) and/or anthropogenic disturbances (Enochs & Manzello, 2012), which can affect the underwater landscape and, specifically the presence of rubble (Wheeler et al., 2005). It is worth noting that both Maeganeku1 and 2 are located in the proximities of a dredged channel, which may have an effect on rubble size and smoothness/complexity. Both of these parameters are known to affect habitat availability and, consequently, cryptofauna abundance and diversity (Kostylev et al., 2005).

Sites with a higher frequency of “branching–tabular” rubble shapes were associated with higher levels of abundance and diversity. These results are in line with previous research that attributed differences in density of coral reef cryptofauna with the spatial complexity of the associated coral frameworks, either living or dead (Enochs, 2011). Rubble shape is therefore important in determining total abundance and diversity of cryptofauna at the phylum level. In Okinawa Island, corals have been transplanted by private entities for decades (including fishermen and diving centers; Okubo & Onuma, 2015) and, due to the lack of a centralized coordination until 2011, it is difficult to track whether appropriate assessments were made or not for different sites.

Seasonal effects were not tested as a factor in this study, due to the insufficient number of replicates (two winter seasons, one summer season). However, the analyses conducted within the same season (only summer replicates, only winter replicates) gave similar results to those of the full dataset. Therefore, seasonal effects, if present, do not seem to lead to different conclusions when evaluating the present situation using coral rubble cryptofauna. Even considering only the winter 2019 dataset, the differences in animal abundances between control and outplanted locations were not significant. As monitoring of these sites will continue season by season over the coming years, additional data will be collected and allow to better define the role of seasonal variation in the abundance and diversity of rubble cryptofauna.

### Rubble cryptofauna vs. living coral community

The Site factor had a significant effect on both living coral coverage and coral rubble cryptofauna. However, while Manza had the highest coral and *Acropora* coverage, Maeganeku1 was the site with the highest rubble cryptofauna abundance. The Manza site, although having the highest coverage of *Acropora* (10.5%), had a lower frequency of branching-tabular coral rubble, which may have been related to the healthier state of branching corals at this site, particularly *Acropora*. Both the data from rubble cryptofauna and from the living coral community (= coral coverage) highlighted how differences between different sites are larger than those between treatments (outplanted vs. control locations). Cryptofauna showed no differences between outplanted locations and controls, while coral coverage was lower at outplanted locations.

Studies at the phylum level are suitable in describing ecosystem diversity and environmental impacts: Anderson et al. (2005) measured biodiversity (richness, total abundance and structural composition) of animals living in the kelp holdfasts considering major phyla community. Other applications of phylum-level diversity data include the study of human impacts or interventions on natural ecosystems. The loss of fine-scale species information is compensated by a reduction in noise variability from lower taxonomic levels, making this level suitable to monitor ecosystem responses to human intervention (Warwick, 1993). Another key advantage is speed: monitoring at high taxonomic levels allows obtaining the results of a survey in shorter times, which is important when monitoring where considerable public investments are made. Further advantages of using phylum-level data include reduced operative costs (studies which use phylum-level identification can be up to 95% less expensive than those which employ species-level identification) for sorting and identification (Khan, 2006). Taxonomic sufficiency of phylum-level data has been confirmed for a wide range of studies, for terrestrial (Souza et al., 2016), freshwater (Cabral et al., 2017) and marine (Anderson et al., 2005) ecosystems.

In summary, live coral coverage and rubble data agreed in demonstrating the different environmental situations between sites. The two datasets can be considered complementary, as the information they give integrate each other at different spatial scales and for different components of the coral reef community. Sampling coral rubble can provide a variety of data including abundances and diversity of cyptofaunal groups that are often overlooked when dealing with reef restoration, although rubble fauna data have been used before to study biodiversity in different environments (Takada et al., 2014) as well as in coral nurseries (Wee et al., 2019).

## Conclusions

Overall, our data showed that, as of 2019, living coral cover was below 10% at two of the three examined sites, and that coverage was always lower at outplanted locations. As for rubble cryptofauna, abundances at the three outplanted locations were comparable with those of control locations, indicating no obvious short-term changes resulting from the coral outplant operations. As coral reef recovery often requires long times (up to decades) to manifest (Pearson, 1981; Connell, Hughes & Wallace, 1997; Graham et al., 2015), eventual positive effects will need to be evaluated in additional future surveys, and the present study represents a methodological foundation and a baseline dataset for such future work. Future monitoring will assess eventual increases in cover at longer time scales over the current baseline, but a full recovery will be hard to assess given the lack of pre-impact data.

At the same time, several initiatives could be undertaken to reduce stressors that notably increase recovery times (Connell, 1997). Currently, fishing has not been restricted at the outplanted locations. Fish are known to have important roles in reef resilience (Cramer et al., 2017; Kuempel & Altieri, 2017), and as well the act of fishing can negatively impact the coral community. As an example, at the surveyed locations, and especially in Maeganeku (1 and 2) we observed numerous damages to the benthic environment caused by anchors, fishing lines, and nets. Protecting the locations where restoration is underway could significantly help the coral community to recover, perhaps even independently from outplanting and restoration efforts. Especially when restoration results are still uncertain, it is important to associate outplanting activities with integrated management, including protection and conservation. As well, fishing activities are known to change the trophic structure of coral reef fishes in the Pacific (Ruppert et al., 2018). Managing fishing pressure might help increasing the number of fishes, while at the same time helping to protect corals from further damage, with potential positive effects on the benthic community across multiple levels of the trophic net (Cesar, 2000; Topor et al., 2019).

Notably, the fact that the Okinawa Prefectural Government is working on improving the status of its coral reefs is without question a positive development, and the science from this project can help improve future restoration efforts. However, transplanted colonies are subject to the same impacts and stress factors as pre-existing native colonies. Because reefs of Okinawa Island have been affected by decades of anthropogenic impacts, and natural coral populations have been decreasing (Nakano, 2004a; Hongo & Yamano, 2013; Kayanne, Suzuki & Liu, 2017; Masucci & Reimer, 2019), we recommend that Okinawa Prefecture identifies areas where corals are still relatively healthy and apply stricter protection and conservation regulations and more detailed monitoring protocols. It is important to remember that restoration, even when successful, has been estimated to provide ecosystem benefits estimated to be around six orders of magnitude lower than the amount of damage that occurs (Okubo & Onuma, 2015), and thus protection of existing healthy reefs should take priority over the restoration of damaged reefs (Abelson, 2006). Coastal construction and reclamation have been considered as the primary cause of coral mortality in Okinawa (Nakano, 2004b), and coastal development and construction are ongoing, even in Onna Village (e.g., new parking lots and shopping mall under construction at Cape Manzamo, Onna Village in 2019). By combining restoration efforts with more effective environmental protection and more environmentally-aware development practices, Okinawa may be able to conserve its remaining coral reefs.

## Supplemental Information

10.7717/peerj.9185/supp-1 Supplemental Information 1 Example of sampled coral rubble.

10.7717/peerj.9185/supp-2 Supplemental Information 2 Specimen from rubble cryptofauna. Scale = 1 mm.

10.7717/peerj.9185/supp-3 Supplemental Information 3 Raw Data.

## Additional Information and Declarations

### Field Study Permissions

The following information was supplied relating to field study approvals (i.e., approving body and any reference numbers): Fieldwork was done in collaboration with the Okinawa Prefectural Government (environmental division). No approval was required in the prefecture for this kind of survey.

### Data Availability

The following information was supplied regarding data availability: The raw measurements of rubble cryptofauna and coral community cover are available in the Supplemental Files.

## References

[ref-1] Abelson A (2006). Artificial reefs vs coral transplantation as restoration tools for mitigating coral reef deterioration: benefits, concerns, and proposed guidelines. Bulletin of Marine Science.

[ref-2] Anderson MJ (2001). A new method for non-parametric multivariate analysis of variance. Austral Ecology.

[ref-3] Anderson MJ, Diebel CE, Blom WM, Landers TJ (2005). Consistency and variation in kelp holdfast assemblages: spatial patterns of biodiversity for the major phyla at different taxonomic resolutions. Journal of Experimental Marine Biology and Ecology.

[ref-4] Babcock RC (1985). Growth and mortality in juvenile corals (*Goniastrea, Platygyra* and *Acropora*): the first year. Proceedings of the 5th International Coral Reef Congress, Tahiti.

[ref-5] Bartlett MS (1937). Properties of sufficiency and statistical tests. Proceedings of the Royal Society of London Series A.

[ref-6] Beenaerts N, Berghe EV (2005). Comparative study of three transect methods to assess coral cover, richness and diversity. Western Indian Ocean Journal of Marine Science.

[ref-7] Cabral AF, Buosi PRB, Segóvia BT, Velho LFM, Bini LM (2017). Taxonomic sufficiency in detecting hydrological changes and reproducing ordination patterns: a test using planktonic ciliates. Ecological Indicators.

[ref-8] Casey JM, Connolly SR, Ainsworth TD (2015). Coral transplantation triggers shift in microbiome and promotion of coral disease associated potential pathogens. Scientific Reports.

[ref-75] Cesar HS (2000). Coral reefs: their functions, threats and economic value. Collected Essays on the Economics of Coral Reefs.

[ref-9] Choi DR (1984). Ecological succession of reef cavity-dwellers (coelobites) in coral rubble. Bulletin of Marine Science.

[ref-10] Connell JH (1997). Disturbance and recovery of coral assemblages. Coral Reefs.

[ref-11] Connell JH, Hughes TP, Wallace CC (1997). A 30-year study of coral abundance, recruitment, and disturbance at several scales in space and time. Ecological Monographs.

[ref-12] Cramer KL, O’Dea A, Clark TR, Zhao JX, Norris RD (2017). Prehistorical and historical declines in Caribbean coral reef accretion rates driven by loss of parrotfish. Nature Communications.

[ref-13] Edwards AJ, Clark S (1999). Coral transplantation: a useful management tool or misguided meddling?. Marine Pollution Bulletin.

[ref-14] English S, Wilkinson C, Baker V (1997). Survey manual for tropical marine resources.

[ref-15] Enochs IC (2011). Motile cryptofauna associated with live and dead coral substrates: implications for coral mortality and framework erosion. Marine Biology.

[ref-16] Enochs IC, Manzello DP (2012). Responses of cryptofaunal species richness and trophic potential to coral reef habitat degradation. Diversity.

[ref-17] Fraser SB, Sedberry GR (2008). Reef morphology and invertebrate distribution at continental shelf edge reefs in the South Atlantic Bight. Southeastern Naturalist.

[ref-18] Fujii T, Reimer JD (2011). Phylogeny of the highly divergent zoanthid family Microzoanthidae (Anthozoa, Hexacorallia) from the Pacific. Zoologica Scripta.

[ref-19] Gabriel KR (1971). The biplot graphical display of matrices with application to principal component analysis. Biometrika.

[ref-20] Gilmour JP, Smith LD, Heyward AJ, Baird AH, Pratchett MS (2013). Recovery of an isolated coral reef system following severe disturbance. Science.

[ref-21] Graham NA, Jennings S, MacNeil MA, Mouillot D, Wilson SK (2015). Predicting climate-driven regime shifts versus rebound potential in coral reefs. Nature.

[ref-22] Graham NAJ, Nash KL (2013). The importance of structural complexity in coral reef ecosystems. Coral Reefs.

[ref-23] Harriott VJ (1985). Recruitment patterns of scleractinian corals at Lizard island, Great Barrier Reef. Proceedings of the 5th International Coral Reef Symposium.

[ref-24] Higa Y, Omori M (2014). Production of coral colonies for outplanting using a unique rearing method of donor colonies at Onna Village, Okinawa, Japan. Galaxea, Journal of Coral Reef Studies.

[ref-25] Higa Y, Shinzato C, Zayasu Y, Nagata T, Nakamura R, Yokokura A, Janadou S, Omori M (2018). Flexible development of techniques for coral reef restoration using asexual reproduction in the coral reef preservation and rehabilitation project by Okinawa prefectural government, Japan. Journal of the Japanese Coral Reef Society.

[ref-26] Hoeksema BW, Renema W (2007). Delineation of the Indo-Malayan centre of maximum marine biodiversity: the coral triangle. Biogeography, Time, and Place: Distributions, Barriers, and Islands.

[ref-27] Hollander M, Wolfe DA, Chicken E (2013). Nonparametric statistical methods.

[ref-28] Hongo C, Yamano H (2013). Species-specific responses of corals to bleaching events on anthropogenically turbid reefs on Okinawa Island, Japan, over a 15-year period (1995–2009). PLOS ONE.

[ref-29] Huang D, Benzoni F, Fukami H, Knowlton N, Smith ND, Budd AF (2014). Taxonomic classification of the reef coral families Merulinidae, Montastraeidae, and Diploastraeidae (Cnidaria: Anthozoa: Scleractinia). Zoological Journal of the Linnean Society.

[ref-30] Japan Statistics Bureau (2014). Japan statistical yearbook 2014. 1-1 Islands, area and major islands of national land. https://www.stat.go.jp/english/data/nenkan/68nenkan/1431-01.html.

[ref-31] Kamezaki M, Higa M, Hirose M, Suda S, Reimer JD (2013). Different zooxanthellae types in populations of the zoanthid *Zoanthus sansibaricus* along depth gradients in Okinawa, Japan. Marine Biodiversity.

[ref-32] Kayanne H, Suzuki R, Liu G (2017). Bleaching in the Ryukyu Islands in 2016 and associated degree heating week threshold. Galaxea, Journal of Coral Reef Studies.

[ref-33] Kendall MG (1938). A new measure of rank correlation. Biometrika.

[ref-34] Khan SA (2006). Is species level identification essential for environmental impact studies?. Current Science.

[ref-35] Kobluk DR, Lysenko MA (1987). Impact of two sequential Pacific hurricanes on sub-rubble cryptic corals: the possible role of cryptic organisms in maintenance of coral reef communities. Journal of Paleontology.

[ref-36] Kostylev VE, Erlandsson J, Mak YM, Williams GA (2005). The relative importance of habitat complexity and surface area in assessing biodiversity: fractal application on rocky shores. Ecological Complexity.

[ref-37] Kramer MJ, Bellwood O, Bellwood DR (2013). The trophic importance of algal turfs for coral reef fishes: the crustacean link. Coral Reefs.

[ref-38] Kuempel CD, Altieri AH (2017). The emergent role of small-bodied herbivores in pre-empting phase shifts on degraded coral reefs. Scientific Reports.

[ref-39] Loya Y, Sakai K, Yamazato K, Nakano Y, Sambali H, van Woesik R (2001). Coral bleaching: the winners and the losers. Ecology Letters.

[ref-40] Masucci GD, Biondi P, Negro E, Reimer JD (2019). After the long summer: death and survival of coral communities in the shallow waters of Kume island, from the Ryukyu archipelago. Regional Studies in Marine Science.

[ref-41] Masucci GD, Reimer JD (2019). Expanding walls and shrinking beaches: loss of natural coastline in Okinawa Island, Japan. PeerJ.

[ref-42] Mori M (1995). Movement of stony corals and crown-of-thorns starfish in Sekisei Lagoon. Marine Parks Journal.

[ref-43] Nakano Y, Ministry of the Environment and Japanese Coral Reef Society (2004a). Global environmental change and coral bleaching. Coral Reefs of Japan (Chapter 2).

[ref-44] Nakano Y, Ministry of the Environment and Japanese Coral Reef Society (2004b). Direct impacts of coastal development. Coral Reefs of Japan (Chapter 2).

[ref-45] Nishihira M, Ministry of the Environment and Japanese Coral Reef Society (2004). Hermatypic corals of Japan. Coral Reefs of Japan (Chapter 1).

[ref-46] Okinawa Prefectural Government (2017). Coral reef conservation and regeneration project report, chapter 2-1. https://www.pref.okinawa.jp/site/kankyo/shizen/hogo/documents/sangohosaisoukatsu2-1.pdf.

[ref-47] Okinawa Prefectural Government (2019). Population estimates. https://www.pref.okinawa.jp/toukeika/estimates/estimates_suikei.html.

[ref-48] Okubo N, Onuma A (2015). An economic and ecological consideration of commercial coral transplantation to restore the marine ecosystem in Okinawa, Japan. Ecosystem Services.

[ref-49] Omori M (2011). Degradation and restoration of coral reefs: experience in Okinawa, Japan. Marine Biology Research.

[ref-50] Omori M, Higa Y, Shinzato C, Zayasu Y, Nagata T, Nakamura R, Yokokura A, Janadou S (2016). Development of active restoration methodologies for coral reefs using asexual reproduction in Okinawa, Japan.

[ref-51] Pearson RG (1981). Recovery and recolonization of coral reefs. Marine Ecology Progress Series.

[ref-52] R Development Core Team (2019). R: a language and environment for statistical computing.

[ref-53] Rasser M, Riegl B (2002). Holocene coral reef rubble and its binding agents. Coral Reefs.

[ref-54] Reimer JD, Biondi P, Lau YW, Masucci GD, Nguyen XH, Santos MEA, Wee HB (2019). Marine biodiversity research in the Ryukyu Islands, Japan: current status and trends. PeerJ.

[ref-55] Reimer JD, Yang S-Y, White KN, Asami R, Fujita K, Hongo C, Ito S, Kawamura I, Maeda I, Mizuyama M, Obuchi M, Sakamaki T, Tachihara K, Tamura M, Tanahara A, Yamaguchi A, Jenke-Kodama H (2015). Effects of causeway construction on environment and biota of subtropical tidal flats in Okinawa, Japan. Marine Pollution Bulletin.

[ref-56] Richter C, Wunsch M, Rasheed M, Kötter I, Badran MI (2001). Endoscopic exploration of Red Sea coral reefs reveals dense populations of cavity-dwelling sponges. Nature.

[ref-57] Royston P (1982). Algorithm AS 181: the W test for normality. Applied Statistics.

[ref-58] Ruppert JL, Vigliola L, Kulbicki M, Labrosse P, Fortin MJ, Meekan MG (2018). Human activities as a driver of spatial variation in the trophic structure of fish communities on Pacific coral reefs. Global Change Biology.

[ref-59] Sakai K, Nishihira M, Nishihira M (1986). Ecology of hermatypic corals. Coral reefs of Okinawa.

[ref-60] SER (2004). The SER international primer on ecological restoration: society for ecological restoration international, Tuscon, Arizona. www.ser.org.

[ref-61] Soong K, Chen TA (2003). Coral transplantation: regeneration and growth of *Acropora* fragments in a nursery. Restoration Ecology.

[ref-62] Souza JLP, Baccaro FB, Landeiro VL, Franklin E, Magnusson WE, Pequeno PACL, Fernandes IO (2016). Taxonomic sufficiency and indicator taxa reduce sampling costs and increase monitoring effectiveness for ants. Diversity and Distributions.

[ref-63] Takada Y, Abe O, Shibuno T (2007). Colonization patterns of mobile cryptic animals into interstices of coral rubble. Marine Ecology Progress Series.

[ref-64] Takada Y, Ikeda H, Hirano Y, Saigusa M, Hashimoto K, Abe O, Shibuno T (2014). Assemblages of cryptic animals in coral rubble along an estuarine gradient spanning mangrove, seagrass, and coral reef habitats. Bulletin of Marine Science.

[ref-65] Topor ZM, Rasher DB, Duffy JE, Brandl SJ (2019). Marine protected areas enhance coral reef functioning by promoting fish biodiversity. Conservation Letters.

[ref-66] Troyer EM, Coker DJ, Berumen ML (2018). Comparison of cryptobenthic reef fish communities among microhabitats in the Red Sea. PeerJ.

[ref-67] Van Woesik R, Sakai K, Ganase A, Loya Y (2011). Revisiting the winners and the losers a decade after coral bleaching. Marine Ecology Progress Series.

[ref-68] Veron JEN (2000). Corals of the World.

[ref-69] Vytopil E, Willis B (2001). Epifaunal community structure in *Acropora* spp. (Scleractinia) on the Great Barrier Reef: implications of coral morphology and habitat complexity. Coral Reefs.

[ref-70] Wallace CC (1983). Visible and invisible coral recruitment.

[ref-71] Warwick RM (1993). Environmental impact studies on marine communities: pragmatical considerations. Australian Journal of Ecology.

[ref-72] Wee SYC, Sam SQ, Sim WT, Ng CSL, Taira D, Afiq-Rosli L, Kikuzawa YP, Toh TC, Chou LM (2019). The role of in situ coral nurseries in supporting mobile invertebrate epifauna. Journal for Nature Conservation.

[ref-73] Wheeler AJ, Bett BJ, Billett DSM, Masson DG, Mayor DJ (2005). The impact of demersal trawling on northeast Atlantic deepwater coral habitats: the case of the Darwin mounds. United Kingdom. American Fisheries Society Symposium.

[ref-74] Yandell BS (1997). Practical data analysis for designed experiments.

