# Supplementary figures and images for: Coral cover and rubble cryptofauna abundance and diversity at outplanted reefs in Okinawa, Japan
Source: PeerJ. 2020 Sep 22;8:e9185. doi: 10.7717/peerj.9185 (PMC7518162; doi:10.7717/peerj.9185)

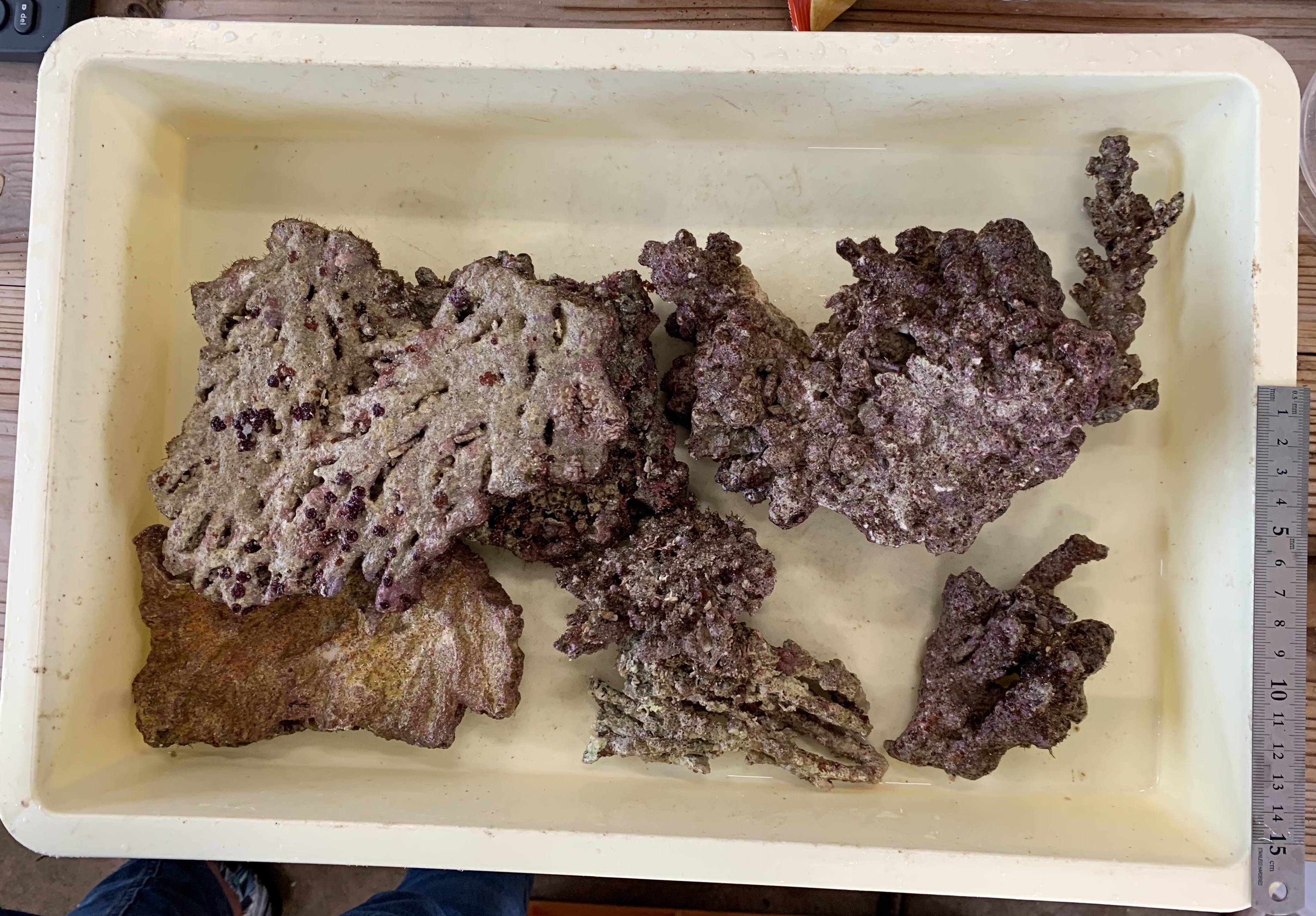

Supplement: Supplemental Information 1 [file peerj-08-9185-s001.jpeg]

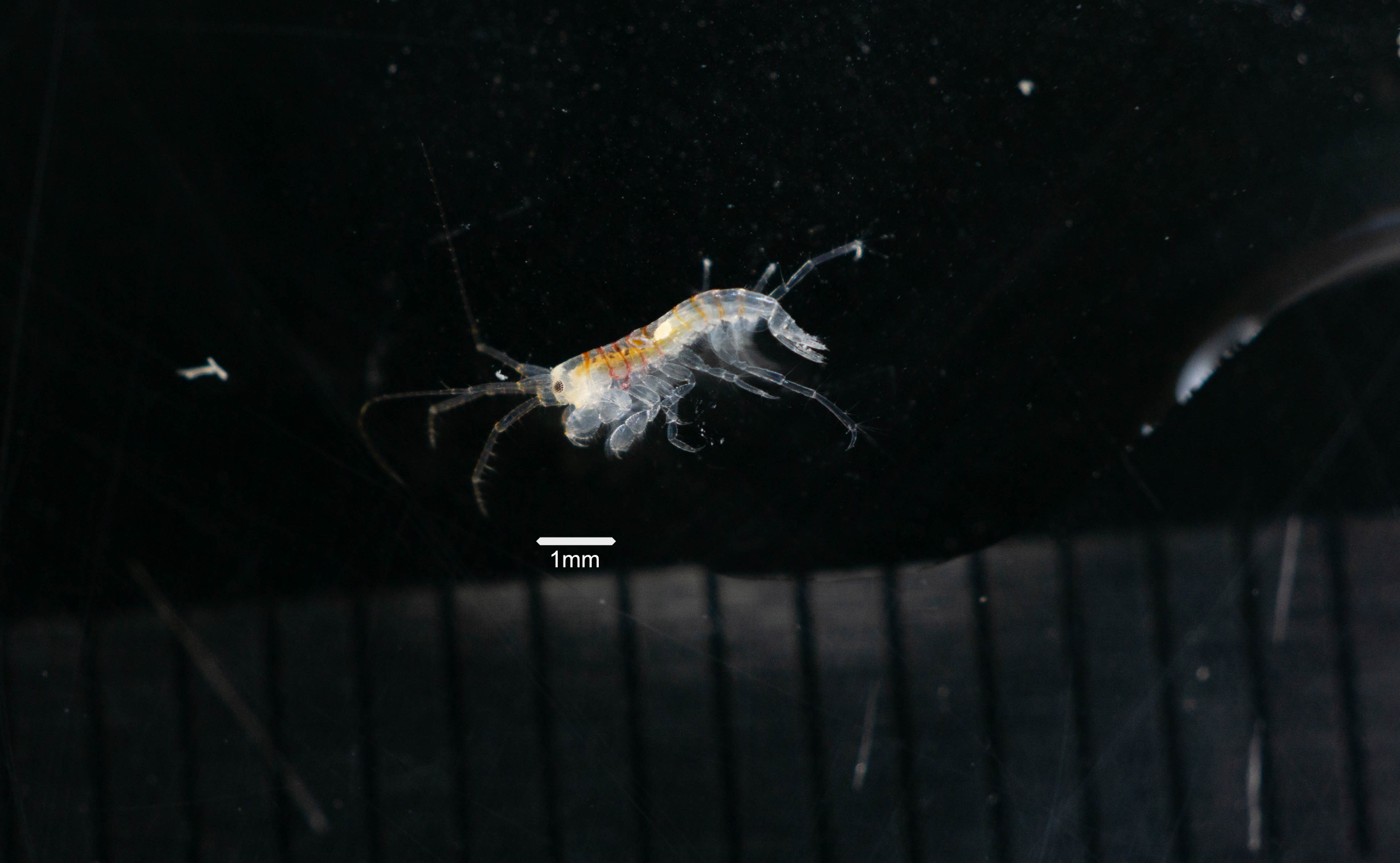

Supplement: Supplemental Information 2 — Scale = 1 mm. [file peerj-08-9185-s002.jpeg]
